# The abstracts of IAP meetings will not be covered in JAPID

**DOI:** 10.34172/japid.2020.008

**Published:** 2020-05-20

**Authors:** Adileh Shirmohammadi

**Affiliations:** ^1^Editor-in-Chief, Journal of Advanced Periodontology & Implant Dentistry; ^2^Professor, Department of Periodontics, Faculty of Dentistry, Tabriz University of Medical Sciences, Tabriz, Iran

*Journal of Advanced Periodontology & Implant Dentistry* (JAPID) is the official biannual publication of the Iranian Association of Periodontology (IAP), dedicated to the current and advanced topics in the field of periodontology and dental implants.^1^

The Iranian Association of Periodontology holds regular annual meetings for periodontists and other dental professionals to present, discuss, and disseminate current academic research. Abstracts from a number of these meetings have been published in JAPID. The majority of abstract-based papers have been published in a supplementary issue by the time of their presentation in the meeting.^2,3^

An abstract is basically a research note or a summary of results and seldom does an abstract present sufficient material to be considered a publication. Nonetheless, abstracts are useful in transferring information. Without the supportive paper, abstracts indicate that some research was undertaken, with brief findings, but it did not end up in publication. Peer-reviewed extended abstracts are usually considered publications after they undergo a routine publication process.

Many publications are also shortened to the extent that not all the details or information is presented. In applying for a technical position or undergoing a review of achievements, one might include publications and abstracts, presentations, and the like. University professors and experienced researchers applying for positions or technical work reviews probably observe higher standards and demands for peer-reviewed published works. When abstracts, extended abstracts, and presentations are listed, they should be appropriately noted, and should not be given the same level of importance as peer-reviewed publications. Besides, there are various levels of publication quality, peer review, importance, and difficulty that could be reviewed and rated. Few want to spend time on compiling and rating the works of others. Therefore, whether accomplished or not, it is critical to properly qualify, cite, and present work products for what they are.

In conferences and scientific meetings, the scientific community commonly disseminates knowledge by either oral/poster presentations, or workshops/panel sessions. They are presentations with no requirement for being published or distributed in the form of a written paper. However, some oral presentations undergo full transcript/paper coverage in the conferences. Sometimes merely the abstract or the title appears in the indexed journal. As a result, it is not considered a publication due to a lack of real handling of knowledge. In addition, many conference proceedings often do not get cited. The editorial board of JAPID has, therefore, decided to put an end to publishing IAP meeting abstracts in JAPID, and all the abstracts accepted for presentation will be presented on the conference website before the conference.

## Competing interests

The author(s) declare that they have no competing interests.
